# Generalization on Both Sides of a Mutualism: Pollinators of *Jacquemontia curtisii* in Southern Florida

**DOI:** 10.3390/plants14071041

**Published:** 2025-03-27

**Authors:** Suzanne Koptur

**Affiliations:** Department of Biological Sciences, International Center for Tropical Botany, Institute of the Environment, Florida International University, Miami, FL 33199, USA; kopturs@fiu.edu; Tel.: +1-305-984-0539

**Keywords:** clustervine, flowers, generalists, pollen, pollen/ovule ratio, pollinators, stigmas, visitors

## Abstract

*Jacquemontia curtisii* Peter ex Hallier f. is common in the pine rocklands of the southern part of peninsular Florida, with its white star-shaped flowers open to visits from many species of arthropods. Its flowers are visited by a wide array of insects, especially Hymenoptera and Lepidoptera. To determine if any of these flower visitors are specialized to *J. curtisii*, we observed visitors to the flowers of this species, catching visitors and sampling the pollen from their bodies. We examined stigmas of *J. curtisii* from 12 different sites to see how many plant species’ pollen was found and the size of the pollen loads. Though it seemed like many insects were visiting *J. curtisii* exclusively when it was in bloom, a surprising number had pollen of two or more other co-occurring plant species, indicating that the flower-visiting bees were generalists, as were the flowers of *Jacquemontia curtisii*. We compared the list of flower visitors with those observed at two previously studied southern Florida *Jacquemontia* species, *J. pentanthos* (Jacq.) G. Don and *J. reclinata* House ex Small, and compared pollen/ovule ratios of the three species, making predictions about the breeding systems of *J. curtisii* and *J. pentanthos*, as their P/Os are larger than those of *J. reclinata*, which was shown previously to be mostly self-incompatible.

## 1. Introduction

Festooned with bright, star-shaped flowers on herbaceous stems, climbing over other plants or along the ground, *Jacquemontia* (Convolvulaceae) are regarded among the loveliest and most distinctive flowering plants of coastal dunes, pine rocklands, and scrub habitats in southern Florida. Their wide, open flowers offer both nectar and pollen rewards that are easily accessible to a variety of visitors.

The pollination of members of the Convolvulaceae has been studied worldwide, and the morphology and colors of the flowers are often predictive of the main visitors. Species with white- or light-colored flowers and long floral tubes are visited mainly by hawkmoths [1,2,3,4,5], though some are visited by bats [6]. Convolvulaceae species with red flowers and long floral tubes are visited by hummingbirds [7,8,9] and may provide nectar for others with long mouthparts, such as butterflies, though these may be less important as pollinators [3,7]. Blue, pink, and purple flowers with more open corollas are favored by bees [10,11,12], as are white flowers opening in the morning [13,14]. Some purple flowers open pre-dawn and are visited by hawkmoths as well as bees when they are open in the morning [15]. Though albino flowers of some flowering plant species are visited less than their purple or blue counterparts, this is not the case for some morning glories. Some species open flowers at more than one time in a day, taking advantage of both diurnal and nocturnal visitors [16], as can others that open their flowers from midnight throughout the morning [17].

Many species in this family are self-compatible and show pollinator-dependency, with more fruits produced when flowers are visited [12,18]. Some self-compatible species undergo self-pollination when the flower finally closes [19], as experimentally demonstrated [20], with the assurance of pollen deposition even if the flower is not visited. Additionally, some species close their flowers more quickly when they are pollinated [21]. Self-compatible species of *Ipomoea* have smaller flowers and fewer rewards and attract fewer visitors than self-incompatible species but produce more fruit than self-incompatible ones [17,22]. Although many weedy flowering plants are self-compatible, the weedy *Ipomoea cairica* is self-incompatible [11].

Studies of *Jacquemontia* spp. are fewer than those of larger genera such as *Ipomoea*, but most species that have been examined are self-compatible [14,23,24,25]. As self-compatible plants are able to also set fruit with other individuals, it is no surprise that some self-compatible species show greater reproductive output with cross-pollination: *Jacquemontia montana* was predicted to be facultatively xenogamous based on its pollen/ovule ratio [26], *J. multiflora* produces twice as much fruit with cross-pollination as self-pollination [23], and *J. reclinata* cross-pollinations were more than twice as successful as self-pollinations in setting fruits and had substantially more seeds per fruit as well [27].

The flowers of *Jacquemontia curtisii* Peter ex Hallier f. have open, sympetalous corollas that leave reproductive parts and floral rewards accessible to many sizes and types of visitors. Due to their open morphology, we anticipated a variety of floral visitors and potential pollinators to visit. As they flower abundantly and seasonally, we expected that some visitors might specialize in these flowers, availing themselves of the plant’s nectar and pollen rewards to the exclusion of other co-blooming plant species.

This study sought to answer the following questions: (1) Do the flowers of *Jacquemontia curtisii* interact with a special type of floral visitor? (2) What is the pollen/ovule ratio of *J. curtisii*, and what does it suggest about its breeding system and specialization? (3) Do flower visitors to *J. curtisii* specialize in its flowers? (4) Does the size of the habitat fragment influence the proportion of *J. curtisii* flowers visited, as well as the amount and diversity of pollen deposited by floral visitors? Information about plant reproductive biology is needed for conservation and plant protection, particularly for this species endemic to the pine rocklands of southern Florida, which is a habitat greatly diminished and still threatened by further development.

## 2. Results

### 2.1. Visitor Species Richness

We observed numerous species of insects visiting the flowers of *J. curtisii* (Table 1). Observations were made over all sites; *J. curtisii* yielded 19 species from three different orders: Diptera, Hymenoptera, and Lepidoptera. We compared the list of visitors to those in published and unpublished data of previous studies (Table 1).

Diptera (flies) were observed only by the white-flowered species (*J. curtisii* and *J. reclinata*), as were Lepidoptera (butterflies and skippers). The blue-flowered *J. pentanthos* only reported bee and wasp visitors (Table 1). Only two bee species, *Melissodes communis* and *Nomia* cf. *maneei*, were recorded from all three of the *Jacquemontia* species. Tiny *Dialictus* bees were observed on all three *Jacquemontia* species, though many specimens were not determined by earlier observers to species, and there may have been different *Dialictus* spp. unique to each plant species.

The most abundant and frequent visitors to *Jacquemontia curtisii* were Diptera (flies), Hymenoptera (bees and wasps), and Lepidoptera (butterflies and skippers), and it was the bodies of these that we examined for pollen. Most Hymenoptera examined had pollen on their bodies. On only two fly individuals (one syrphid fly and one bombyliid fly) was *J. curtisii* pollen present. Each of these flies also had the pollen of at least one other plant species on their bodies (Table 2). No pollen was found on the bodies of the few Lepidoptera specimens collected.

The single leafhopper examined had the pollen of three other plants on its body but no *Jacquemontia* pollen. All bees and wasps examined carried *Jacquemontia* pollen, and all but one individual of the tiny sweat bee *Dialictus coreopsis* (Halictidae) carried the pollen of 1–6 other plant species as well.

### 2.2. Pollen/Ovule Ratios

**Figure 1:**
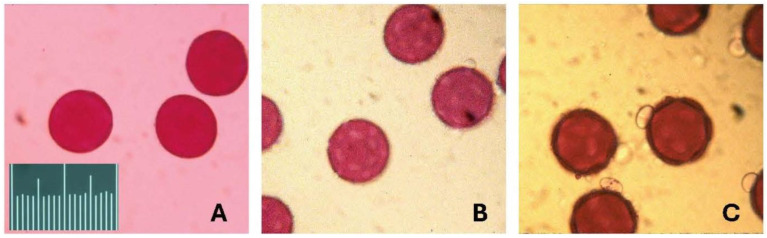
Images of pollen of the three species of *Jacquemontia*. The pollen was mounted in fuchsin gel and photographed under a light microscope at 40× magnification. The pollen grains of these three species are similar in shape and size, all with diameters of 55–60 microns. The scale is 100 microns in units of 5 microns. (**A**) *Jacquemontia curtisii*; (**B**) *J. pentanthos*; and (**C**) *J. reclinata*.

Pollen of the three *Jacquemontia* species is similar in size and shape (Figure 1). Flowers of the three species are also similar in size and, as it turns out, somewhat similar in the ratio of the number of pollen grains per ovule in a flower. Each flower has one style and ovary, and each ovary has four ovules; pollen is produced by five stamens in each flower. We, therefore, counted the number of pollen grains in each of the five flowers (from five different individual plants) of each species, multiplied that number by five to estimate the number of pollen grains per flower, and then divided this by the number of ovules (four).

*Jacquemontia reclinata* has the lowest P/O ratio at around 1600 (1625 +/− 712, range 625–2500); *J. pentanthos* is somewhat greater at 3400 (3375 +/− 2951, range 625–7500); and *J. curtisii* is the highest at around 4000 (4000 +/− 2709, range 625–6875). As the sample sizes here are small (*n* = 5 per species) and the variance among the samples is large, it is not possible to say if these differences are significant among the species (ANOVA non-significant, F = 1.73, (df = 2), *p* = 0.291).

### 2.3. Pollen on Stigmas

**Figure 2:**
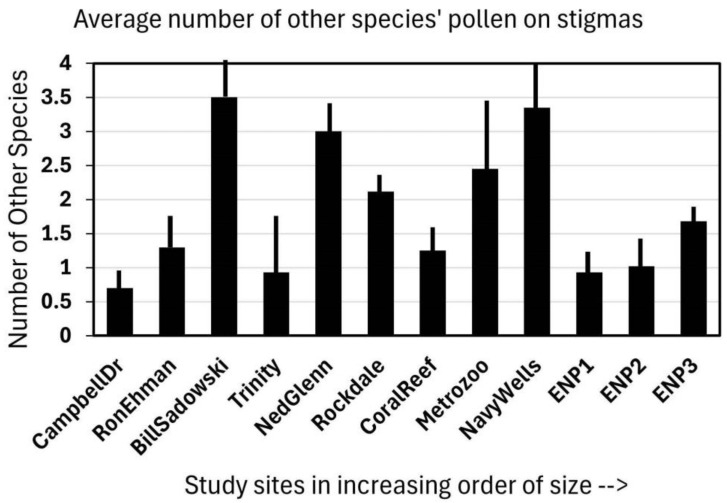
The pollen observed on 15 stigmas of *Jacquemontia curtisii* collected at the end of the flower-opening period (early afternoon) at twelve pine rockland sites along the Miami rock ridge arranged in increasing size order of the natural area. Bars indicate the average number of other species (in addition to *J. curtisii*) from which pollen was found on each stigma collected at each site + the standard deviation.

Observing the stigmas of *Jacquemontia curtisii* flowers that were collected in the early afternoon at 12 sites (in four area-size categories), it was shown that visitors deposited not only the pollen of that species but other species as well. At most of the sites, every flower examined had pollen on its stigma, and although some had only *Jacquemontia* pollen, most had at least two other species deposited (Figure 2). The overall average of all the sites was 1.5 additional species per stigma. As these stigma collections were part of a larger study on habitat fragmentation, the sites had multiple representatives of small, medium, and large pine rockland fragments, as well as three “pristine” sites in Everglades National Park. There was no significant pattern to the amount of additional plant species pollen present on the stigmas; the correlation between site size and the number of additional pollen types found was −0.22.

**Figure 3:**
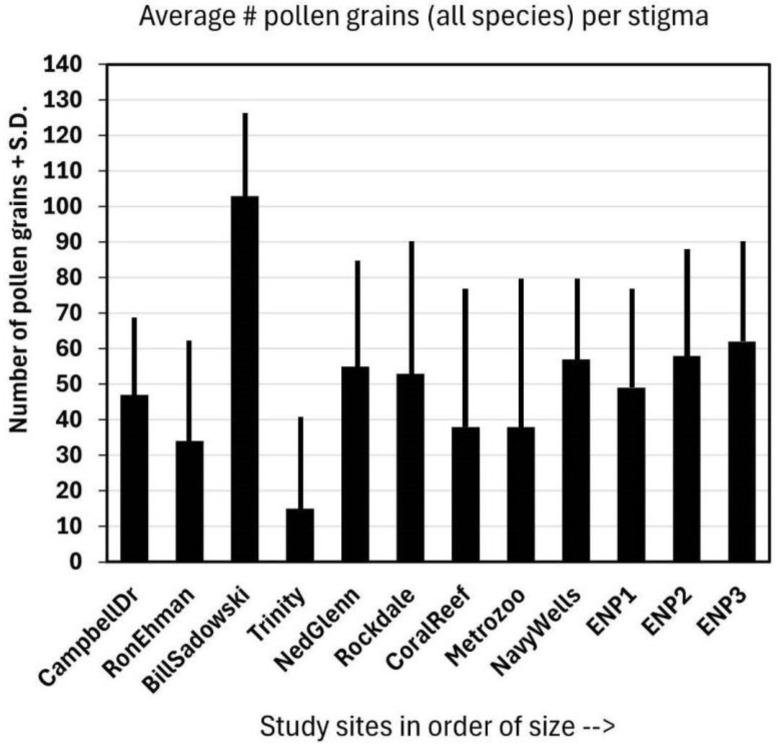
The average of the total number (#) of pollen grains + standard deviation (S.D.) of all species (including *J. curtisii*) on the same 15 stigmas per site as those represented in Figure 2.

The average number of pollen grains deposited on the stigmas of *Jacquemontia curtisii* (Figure 3) at all sites was greater than ten, and the overall average was 25 ± 15. These numbers include flowers that had no pollen on the stigma as well, so those that had pollen (those that had been visited) had even more pollen grains deposited than this average. There appeared to be a trend of greater numbers of pollen grains deposited at larger sites, but one of the smaller fragments (Bill Sadowski) had the largest number of all. The site size was slightly positive, but not significantly, and was correlated with the average pollen load: r = 0.15.

## 3. Discussion

Previous studies on *Jacquemontia* species have shown that most are visited by numerous and diverse visitors [24,25,27], but most have concluded that bees are the most important and effective pollinators [14,26]. Very small bees have been observed to focus on pollen collection [26,27], spending time on the anthers in the proximity of the stigmas and sometimes crawling into the center of the flower to drink nectar. Larger bees often visit for nectar primarily, pushing past the stamens to reach the nectar, and are dusted with pollen in the process.

*Jacquemontia* flowers, like most Convolvulaceae, have “unrestrictive” morphology [28], allowing visitors of many shapes and sizes. Generalization “appears to be the rule rather than the exception” in plant/pollinator interactions [29]. While some members of this family have tubular corollas with flaring petal tips [3,4,6,7,8], restricting access to nectar rewards to only those visitors with mouthparts long enough to reach the sweet liquid pooled at the base of the corolla tube, the nectar of *Jacquemontia* is accessible to all visitors that come to the center of the open corolla. The long-tubed flowers of the more specialized species of Convolvulaceae have their anthers and stigmas exserted so that they are sure to encounter the faces and bodies of visitors probing for nectar, whereas the wide open flowers of *Jacquemontia* have their stamens and styles forward, at right angles to the face of the flower, so that some visitors might be able to access their nectar without encountering the anthers and stigma. Just as the nectar in tubular flowers may be robbed by insects that chew a hole in the base of the corolla tube, the nectar of *Jacquemontia* flowers might be robbed by visitors too small to touch the pollen that supports the structures of the flowers.

The open flowers of *Jacquemontia curtisii* receive visits from numerous species of insects, primarily Hymenoptera, and most visitors carry multiple types of pollen on their bodies. The stigmas of its flowers receive multiple plant species’ pollen as well as its own. From this evidence alone, it appears that this is a generalized mutualism. Pollen loads on stigmas are not correlated with the size of the pine rockland habitat sites, nor is it correlated with the number of different types of pollen deposited. More information is needed about the breeding system (compatibility relationships) of this species to better understand the impact of pollen deposition on plants in smaller versus larger fragments and pristine habitats.

Even visitors that do not fit a certain pollination syndrome may carry pollen, so it is important that in pollination studies, observers document all the visitors from the pollinator’s perspective to learn what other plant species pollinators visit on the same days they visit the plant species of interest. Without following individual visitors from plant to plant, this information can be gleaned by sampling visitors’ bodies of pollen, as we did in this study. As the bodies of bees are hairy in at least some parts, they are ideal vehicles upon which pollen can be inadvertently picked up and carried to other flowers as the bees visit other flowers for nectar; these might be flowers of the same species, or others in the vicinity that also present nectar rewards.

Some bees are pollen collectors and may ignore the nectar rewards of flowers with anthers full of pollen. In Convolvulaceae, with anthers and stigmas in close proximity, their actions may also result in the inadvertent deposition of pollen and pollination of flowers that also have nectar rewards. Pollen is usually collected for the provision of their offspring in nest cells, and so there is another way to indirectly learn what flowers they visit: finding the nests and examining the pollen deposited therein. In the Bahamas, *Megachile* (ground-nesting, solitary bees) use mostly *Corchorus* and *Jacquemontia cayensis* pollen to nourish their nest cells [30], providing evidence from a top-down perspective that these bees visit *J. cayensis* flowers.

Cruden [31] suggested that the ratio of the number of pollen grains to the number of ovules (the P/O ratio) may indicate the breeding system of a plant. More recent analyses [32] have shown that pollinator-dependent plants have more pollen than those that can self-pollinate and that pollination efficiency is also important in terms of how much pollen a plant produces so that P/O ratios overlap for self-compatible and self-incompatible species and produce only a weak correlation with the outcrossing rate. The pollen/ovule ratios of the three species included here are all in the range of what might be expected for plants that facultatively outcross compared to entirely outcrossing species according to Cruden’s scheme; as we conducted our comparison within a genus rather than across clades, the potential differences in the P/O ratios may indicate differences in the degree of self-compatibility among the three species of *Jacquemontia*. The compatibility of the relationships of only one of the species in our study has been previously investigated: we know that in *J. reclinata,* the greater amount of fruit produced results from cross-pollination rather than self-pollination, though some fruit is produced as a result of self-pollination [27]. We predict that the other two species will show greater degrees of self-incompatibility, as their average P/O ratios are greater than those of *J. reclinata*. Hand-pollination experiments are required to test this prediction.

There are drawbacks to generalized flowers, the first of which is the potential wasting of floral rewards (nectar and pollen) in the mouths and on the bodies of visitors that will never deposit any pollen on a conspecific plant. Generalist flowers, therefore, are expected to produce more pollen and have a higher pollen/ovule ratio (P/O) than those with more restrictive floral morphology that limits their visitors to those who can access the concealed rewards.

Another drawback of generalization is that pollen from other plant species can interfere with the right kind of pollen being deposited, a phenomenon dubbed “stigma clogging” [33,34,35,36]. Species with smaller stigmas and lower P/O can be greatly impacted in this way [28,37], as was shown for two sympatric Convolvulaceae species [38]. *Jacquemontia curtisii* fruits have a maximum of four seeds, so the large numbers of pollen grains of other species deposited may preclude compatible *Jacquemontia* pollen from reaching the receptive stigma surface, thwarting ovule fertilization and seed formation. And since this species is self-incompatible, self-pollination can also interfere with compatible pollen from other individuals of the species.

In the pine rocklands of South Florida, individuals of herbaceous plants growing near blooming individuals of palm species may be deprived of visitors that prefer to visit the large concentration of nectar and pollen resources offered by the palms [39]. The palms, rather than serving as “pollinator magnets” to attract visitors to less showy neighboring plants, evidently cause visitors to neglect the smaller plants in favor of the large amounts of nectar and pollen offered by the large inflorescences of the palms. In fact, many of the bee visitors observed at *J. curtisii* flowers also appear in the list of visitors to flowers of the understory sabal and saw palmettos (*Sabal palmetto* and *Serenoa repens*) of the pine rocklands [40]. If the flowers of the herbaceous plants are visited after the bees have visited palm inflorescences, it is likely that palm pollen will be deposited on the stigmas of the herbaceous plants, which is another means of interference competition from palms.

Pine rocklands [41] occur on the more highly elevated land of southern Florida, which is land that is coveted continually for further human development in this heavily populated area. The limestone rock provides solid ground that drains well and is attractive for building upon, and the area of this habitat has been greatly diminished over the last century [42,43]. The proper management of remaining fragments of pine rockland is challenging as it is a fire-dependent successional habitat [44], and Miami-Dade County Natural Areas Management meets these challenges with a strong program of protection, rare species monitoring, exotic plant removal, restoration plantings, and the employment of fire via cooperation with the Department of Forestry when possible. Understanding the pollination and reproductive biology of rare plants in their natural communities [45,46] is important to their conservation and protection [47], and our research is an effort towards this end.

## 4. Materials and Methods

### 4.1. Study Species

**Figure 4:**
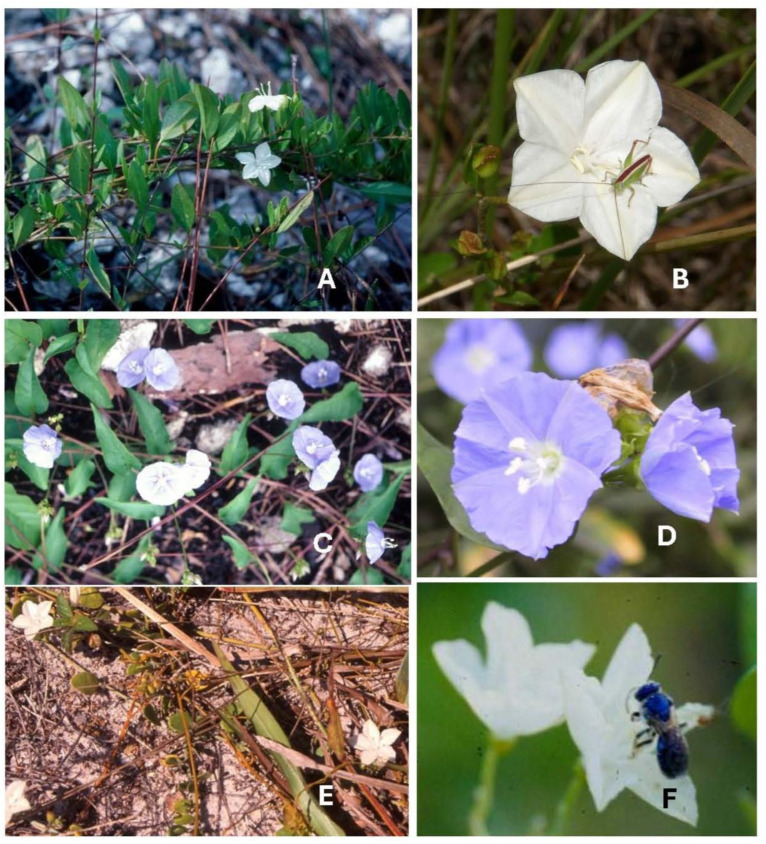
*Jacquemontia* species flower images. (**A**) *Jacquemontia curtisii* in Miami pine rockland; (**B**) *J. curtisii* flower closeup with tiny orthopteran; (**C**) *Jacquemontia pentanthos* in Keys pine rockland; (**D**) *J. pentanthos* flower closeup; (**E**) *Jacquemontia reclinata* in sandy beach coastal strand (D. Bogler photo); and (**F**) *J. reclinata* flower with sweat bee (Halictidae), *Augochloropsis anonyma* (D. Monteith photo).

*Jacquemontia curtisii* Peter ex Hallier f., the pineland clustervine (Figure 4A,B), can be found in Everglades pine rocklands as well as in pine rockland fragments along the Miami rock ridge as it extends northward. It is endemic to South Florida and is listed by the state as threatened [48] due to the great reduction in its habitat by human activities. Pine rocklands occur on higher ground that was developed for building homes, businesses, and agriculture; the loss of this imperiled habitat continues today, as pine rocklands are continually destroyed on private and public lands due to development pressures [49].

*Jacquemontia pentanthos* (Jacq.) G.Don, the sky-blue clustervine (Figure 4C,D), is found in South Florida as well as the Caribbean and Central and South America. In North America, it occurs only in Florida Keys (Monroe County) and is listed by the state as endangered in Florida. This species can be found in pine rocklands and at the edge of hardwood hammocks in the lower Florida Keys. Its beautiful blue flowers and robust vining habit make it popular in native plant landscaping throughout Florida.

*Jacquemontia reclinata* House ex Small, beach clustervine (Figure 4E,F) is endemic to southern peninsular Florida and is found only in sandy soil in coastal strand habitats. It is both state and federally listed as endangered, and its former continuous distribution has been reduced to small populations in protected areas along the highly developed east coast of Florida, in Dade, Broward, and Palm Beach counties [27].

Two other species have been found in southern Florida but are infrequent and were not included in our study as there are no records of visitors to these species: *J. havanensis* (Jacq.)Urb., Havana clustervine—listed as endangered—a white-flowered species common in northern West Indies, rare in Florida, and found only in the Florida Keys; *Jacquemontia tamnifolia* (L.)Griseb.—hairy clustervine, with flowers blue to white in color—found throughout Florida and SE US, formerly *Ipomoea*, growing in white sand scrub, pinelands, and red soil.

### 4.2. Field Methods

**Figure 5:**
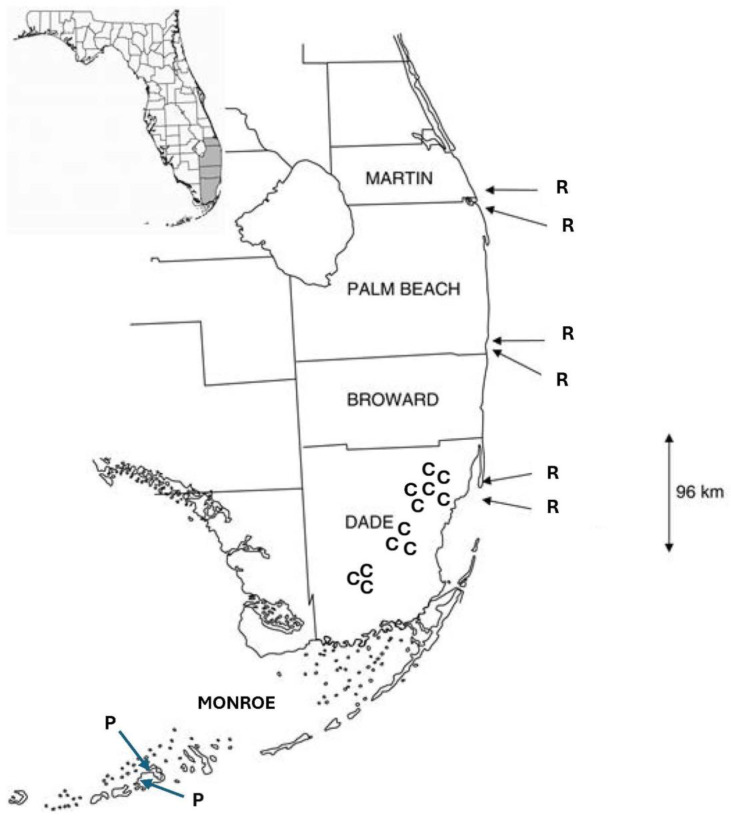
Map of *Jacquemontia* study sites in southern Florida. Counties are labeled in all capital letters, from north to south: Martin, Palm Beach, Broward, Dade, and Monroe. Study sites where each species was studied (previously for the two comparison species) are labeled with the first letter of the specific epithet of each: C = *J. curtisii*; P = *J. pentanthos*; and R = *J. reclinata*.

The fieldwork for this study was conducted only with *Jacquemontia curtisii*. The plants of *J. curtisii* were observed in three areas of pine rocklands in Everglades National Park, where the largest contiguous areas of pine rocklands are found, and in nine pine rockland fragments of different sizes located along the historical extent of this habitat along the Miami rock ridge (Figure 5). The largest fragments and their areas were Navy Wells (99.2 ha), Metrozoo (57.5 ha), and Coral Reef (20.6 ha); the medium-sized fragments were Rockdale (14.7 ha), Ned Glenn (4.6 ha), and Trinity (4 ha); and the small fragments were Bill Sadowski (3 ha), Ron Ehman (0.9 ha), and Campbell Drive (0.4 ha). *Jacquemontia pentanthos* is native to pine rocklands and hammock edges of the Lower Keys, and its flowers were observed in previous studies at several sites: two on Big Pine Key and one on No Name Key. *Jacquemontia reclinata* were previously studied in six sites of coastal strand habitats on the coastline of the southeastern peninsula of Florida [27].

For *Jacquemontia curtisii*, at each site, we observed visitors to the flowers for a total of 30 min, 10 min every half hour over a 90 min period in the morning between 9 am and 11 am. At the end of the observation periods, we captured insect visitors to the flowers with hand-held aerial nets ([50], pp. 263–264) for identification and to sample their bodies for pollen. After capture, each insect was held in individual glassine envelopes to avoid mixing the pollen from different specimens. We examined individual specimens of each visitor species under the dissecting scope, scraping any pollen observed on their bodies onto a glass slide ([41], p. 290) and then placing a drop of melted fuchsin gel ([50], p. 289; [51], p. 107) on the pollen. Fuchsin gel stains viable pollen grains pink and so makes it easier to see under the compound microscope. We sketched all the different types of pollen on each slide, measured the diameters of each type, and counted the number of each pollen type removed from the insect’s body. We later determined the identity of the non-*Jacquemontia* pollen types, but these results are not reported here, as we were concerned primarily by how many species of plants’ pollen were found on each visitor in this study.

We also used observations from earlier studies by other biologists [27], and insect collections left to our lab when Dan Austin retired from Florida Atlantic University to provide a complete list of visitors to the three *Jacquemontia* species. We were not able to sample pollen from those earlier insect collections, so the data of the pollen types reported in this manuscript are only from our field sampling of *J. curtisii*.

As part of a larger study examining the effects of habitat fragmentation on the pollination of pine rockland plants, we obtained data on the pollen on stigmas of *Jacquemontia curtisii* flowers from the same twelve sites (listed previously). We collected stigmas of 15 open flowers of *J. curtisii* at each site and mounted them in fuchsin gel in the field ([50], p. 117) to examine whether the flowers had been visited at protected pine rockland sites of different sizes; we also recorded the numbers of each type of pollen we found on each stigma. This information provided data on the diversity of the types of pollen deposited by floral visitors at the different sites. We examined the slides of mounted stigmas under a compound microscope, recording the numbers of each type of pollen encountered. We used our pollen flora (the collection of images of pollen from known plants) to help determine the species of pollen on the insects and deposited on the stigmas, narrowing our search by considering what other plants were in bloom at the same time. The data presented in this manuscript consist of how many different types of pollen were present in addition to *J. curtisii* pollen and the total number of pollen grains seen on each stigma. We then determined if there was either a positive, negative, or no correlation between site size, the number of pollen types, and the total pollen loads received by each stigma.

Pollen/ovule ratios were calculated from five individuals of each *Jacquemontia* species. A flower bud ready to open the next day was collected, and from it, an anther was removed and placed alone in a small, calibrated sample tube. A set amount (1 mL) of 70% alcohol was added to each sample and shaken until the anther was completely empty of pollen grains. Using a haemocytometer, we counted the number of pollen grains in an aliquot of the solution and multiplied this by the number of aliquots in the sample to estimate the total number in an anther ([51], p. 240). All flowers examined had five stamens, so we multiplied that number by five to obtain the total number of pollen grains per flower. We examined the ovaries of the flowers and found that all ovaries contained four ovules each; we, therefore, divided the total number of pollen grains into five anthers by the number of ovules to obtain the P/O ratio. Means were compared among the three species with univariate ANOVA using IBM SPSS version 29.0.1.1.

## 5. Conclusions

We conclude that although there are many different visitors to each *Jacquemontia* species, the same species of visitor has been recorded visiting more than one *Jacquemontia* species (at different sites as none of these plant species are sympatric). Each *Jacquemontia* species is visited by more than ten species. For *J. curtisii*, the visitors that carry pollen have multiple types of pollen on their bodies, and its stigmas have multiple species of plants’ pollen deposited on them. Both *J. curtisii* plants and the majority of their pollinators are generalists.

In addition, the pollen deposited on the stigmas of *Jacquemontia curtisii* is usually composed of two or more other species in addition to *Jacquemontia* pollen, and the quantities of pollen deposited may interfere with the ability of intraspecific pollen to reach the stigma surface, germinate, and fertilize the ovules. This could be especially important in a likely self-incompatible species like *J. curtisii*, as self-pollination can also prevent compatible cross-pollen from coming in contact with the stigma ([52], pp. 503–504). These findings will direct future experiments in the *J. curtisii* system to better elucidate its breeding system and interactions with other plants in the pine rocklands.

## Figures and Tables

**Table 1:** Visitors to *Jacquemontia curtisii* compared with visitors to two other *Jacquemontia* species studied in South Florida. X indicates the insect was observed visiting flowers of the plant species. Observations were compiled from our fieldwork *on J. curtisii* plus publications and specimens collected on *J. pentanthos* and *J. reclinata* by Elena Pinto-Torres, John Pascarella, Dan Austin, R. A. Mazur, and M. Williams. ** Specimens were damaged, and their identification was not possible.

Order	Family	Insect	*J. curtisii*	*J. pentanthos*	*J. reclinata*
**DIPTERA**	Bombyliidae	*Chrysanthrax cypris* (Meigen 1820)			X
	Dolichopodidae	*Chrysotus* sp.	X		
	Tachinidae	housefly-type fly			X
	Chloropidae	*Liohippelates pusio* Loew, 1872	X		
	Syrphidae	*Ocyptamus fuscipennis* (Say 1823)	X		
	Syrphidae	*Ocyptamus lineatus* (Macquart 1846)	X		
	Syrphidae	*Palpada vinetorum* (Fabricius 1798)			X
	Milichiidae	*Pholeomyia* sp.	X		
	Bombyliidae	*Systoechus* sp.			X
	Syrphidae	*Toxomerus arcifer* (Loew 1866)	X		
	Syrphidae	Unidentified	X		
	Bombyliidae	Unidentified			X
**HETEROPTERA**	Cicadellidae	leafhopper			
	Cicadellidae	leafhopper	X		X
**HYMENOPTERA**	Halictidae	*Agapostemon splendens* (Lepeletier, 1841)		X	X
	Megachilidae	*Anthidium notatum rufimaculatum* Schwarz	X		
	Apidae	*Apis mellifera* L. 1758		X	X
	Halictidae	*Augochlora pura* (Say, 1837)		X	X
	Halictidae	*Augochlorella gratiosa* (Smith, 1853)	X		
	Halictidae	*Augochlorella aurata* Smith, 1853	X		X
	Halictidae	*Augochloropsis anonyma* Cockerell, 1922		X	X
	Vespidae	*Campsomeris plumipes fossulana* (Fabricius)			X
	Vespidae	*Campsomeris trifasciata* (Saussure)	X	X	
	Apidae	*Ceratina cockerelli* (Daly 1973)			X
	Halictidae	*Lasioglossum (Evylaeus) nelumbonis* Robertson			X
	Halictidae	*Lasioglossum (Dialictus) coreopsis* (Robertson)	X		
	Halictidae	*Dialictus* sp.	X	X	X
	Halictidae	*Lasioglossum (Dialictus) tegulare* (Robertson)		X	X
	Halictidae	*Halictus ligatus* Say, 1937	X		X
	Halictidae	*Lasioglossum (Hemihalictus) lustrans* Cockerell		X	X
	Megachilidae	*Megachile parallela* Smith 1853		X	X
	Megachilidae	*Megachile brevis* subsp. *pseudobrevis* Mitchell, 1935			X
	Megachilidae	*Megachile (Sayapis) inimica* Cresson, 1872		X	X
	Megachilidae	*Megachile mendica* Cresson 1878		X	
	Megachilidae	*Megachile* sp. **			X
	Anthophoridae	*Melissodes communis* Cresson, 1878	X	X	X
	Anthophoridae	*Melissoides bimaculata* Lepeletier, 1825			X
	Halictidae	*Nomia* cf. *maneei* Cockerell 1910	X	X	X
	Scoliidae	*Scolia nobilitata* Fabricius, 1805			X
	Vespidae	Unidentified			X
	Apidae	*Xylocopa micans* Lepeletier 1841		X	
**LEPIDOPTERA**	Nymphalidae	*Agraulis vanillae* (L.)	X		X
	Hesperiidae	Unidentified skippers	X		X
**Total visitor species observed per *Jacquemontia* species**			19 spp.	14 spp.	29 spp.

**Table 2:** Pollen on visitor specimens collected visiting *Jacquemontia curtisii* flowers on plants in the field of Miami-Dade County, Florida. # indiv. indicates the number of insect specimens of that taxon examined for pollen; Jacq poll? indicates the presence (yes) or absence (no) of *J. curtisii* pollen; # other spp. indicates the amount of other plant species’ pollen observed on the specimens; and Total spp. indicates the total number of plant species’ pollen on the insect specimen.

Order	Family	Bee ID	# indiv.	Jacq Poll?	# Other spp.	Total spp.
DIPTERA	Bombyliidae	*Chrysanthrax cypris*	1	yes	2	3
	Syrphidae	*Ocyptamus lineatus*	2	no	0	0
	Syrphidae	*Palpada vinetorum*	1	no	3	3
	Syrphidae	Syrphid fly	1	yes	1	2
HETEROPTERA	Cicadellidae	Leafhopper	1	no	3	3
HYMENOPTERA	Apidae	*Apis mellifera*	2	yes	2	3
	Apidae	*Ceratina cockgrelli*	1	yes	2	3
	Apidae	Melissodes communis	3	yes	2–6	3–7
	Apidae	*Xylocopa micans*	1	yes	3	4
	Halictidae	*Augochloropsis anonyma*	1	yes	1	2
	Halictidae	*Augochlorella gratiosa*	5	yes	2–5	3–6
	Halictidae	*Augochloropsis anonyma*	1	yes	1	2
	Halictidae	*Dialictus coreopsis*	2	yes	0–4	1–5
	Halictidae	*Dialictus tegularis*	1	yes	2	3
	Halictidae	*Halictus ligatus*	2	yes	3–6	4–7
	Megachilidae	*Megachile brevis pseudobrevis*	1	yes	2	3
	Megachilidae	*Megachile mendica*	1	yes	4	5
	Scoliidae	*Scolia nobilitata*	2	yes	3–4	4–5
	Vespidae	*Campsomeris trifasciatus*	1	yes	3	4

## Data Availability

The data used in this study will be made freely available upon publication from the FIU Research Data Portal at https://doi.org/10.34703/gzx1-9v95/ATH2BA.
